# A novel theory of Asian elephant high-frequency squeak production

**DOI:** 10.1186/s12915-021-01026-z

**Published:** 2021-06-17

**Authors:** Veronika C. Beeck, Gunnar Heilmann, Michael Kerscher, Angela S. Stoeger

**Affiliations:** 1 Department of Behavioural and Cognitive Biology, Mammal Communication Lab, University of Vienna, Vienna, Austria; 2 gfai tech GmbH, Berlin, Germany

**Keywords:** Communication, Acoustic allometry, Bioacoustics, Vocal signals, Vocal learning, Sound visualization, Functional morphology

## Abstract

**Background:**

Anatomical and cognitive adaptations to overcome morpho-mechanical limitations of laryngeal sound production, where body size and the related vocal apparatus dimensions determine the fundamental frequency, increase vocal diversity across taxa. Elephants flexibly use laryngeal and trunk-based vocalizations to form a repertoire ranging from infrasonic rumbles to higher-pitched trumpets. Moreover, they are among the few evolutionarily distantly related animals (humans, pinnipeds, cetaceans, birds) capable of imitating species-atypical sounds. Yet, their vocal plasticity has so far not been related to functions within their natural communicative system, in part because not all call types have been systematically studied. Here, we reveal how Asian elephants (*Elephas maximus)* produce species-specific squeaks (F0 300–2300 Hz) by using acoustic camera recordings to visualize sound emission and examining this alongside acoustic, behavioral, and morphological data across seven captive groups.

**Results:**

We found that squeaks were emitted through the closed mouth in synchrony with cheek depression and retraction of the labial angles. The simultaneous emission of squeaks with nasal snorts (biphonation) in one individual confirmed that squeak production was independent of nasal passage involvement and this implicated oral sound production. The squeaks’ spectral structure is incongruent with laryngeal sound production and aerodynamic whistles, pointing to tissue vibration as the sound source. Anatomical considerations suggest that the longitudinal closed lips function as the vibrators. Acoustic and temporal parameters exhibit high intra- and inter-individual variability that enables individual but no call-subtype classification. Only 19 of 56 study subjects were recorded to squeak, mostly during alarming contexts and social arousal but some also on command.

**Conclusion:**

Our results strongly suggest that Asian elephants force air from the small oral cavity through the tensed lips, inducing self-sustained lip vibration. Besides human brass players, lip buzzing is not described elsewhere in the animal kingdom. Given the complexity of the proposed mechanism, the surprising absence of squeaking in most of the unrelated subjects and the indication for volitional control, we hypothesize that squeak production involves social learning. Our study offers new insights into how vocal and cognitive flexibility enables mammals to overcome size-related limitations of laryngeal sound production. This flexibility enables Asian elephants to exploit a frequency range spanning seven octaves within their communicative system.

## Background

What makes a brass trumpet sound is first and foremost the player pressing air from puffed out cheeks through closely tensed lips, inducing self-sustained lip oscillation. The lips are periodically forced open and closed by the air pressure and flow interplaying with myoelastic tissue properties—just as in vocal fold sound production [1–3]. The instrument then merely forms the spectral structure by resonating the sound produced by the vibration of the “buzzing lips.” This principle parallels the source-filter theory of vocal production [4, 5], whose application beyond human speech fostered a growing understanding of how morphology and information content covary in animal signals [6]. In humans, the inclusion of non-laryngeal sound sources and the aid of instruments external to the vocal tract, combined with the cognitive capabilities to learn how to use them, clearly multiply the versatility of sounds producible beyond speech.

**Fig. 1:**
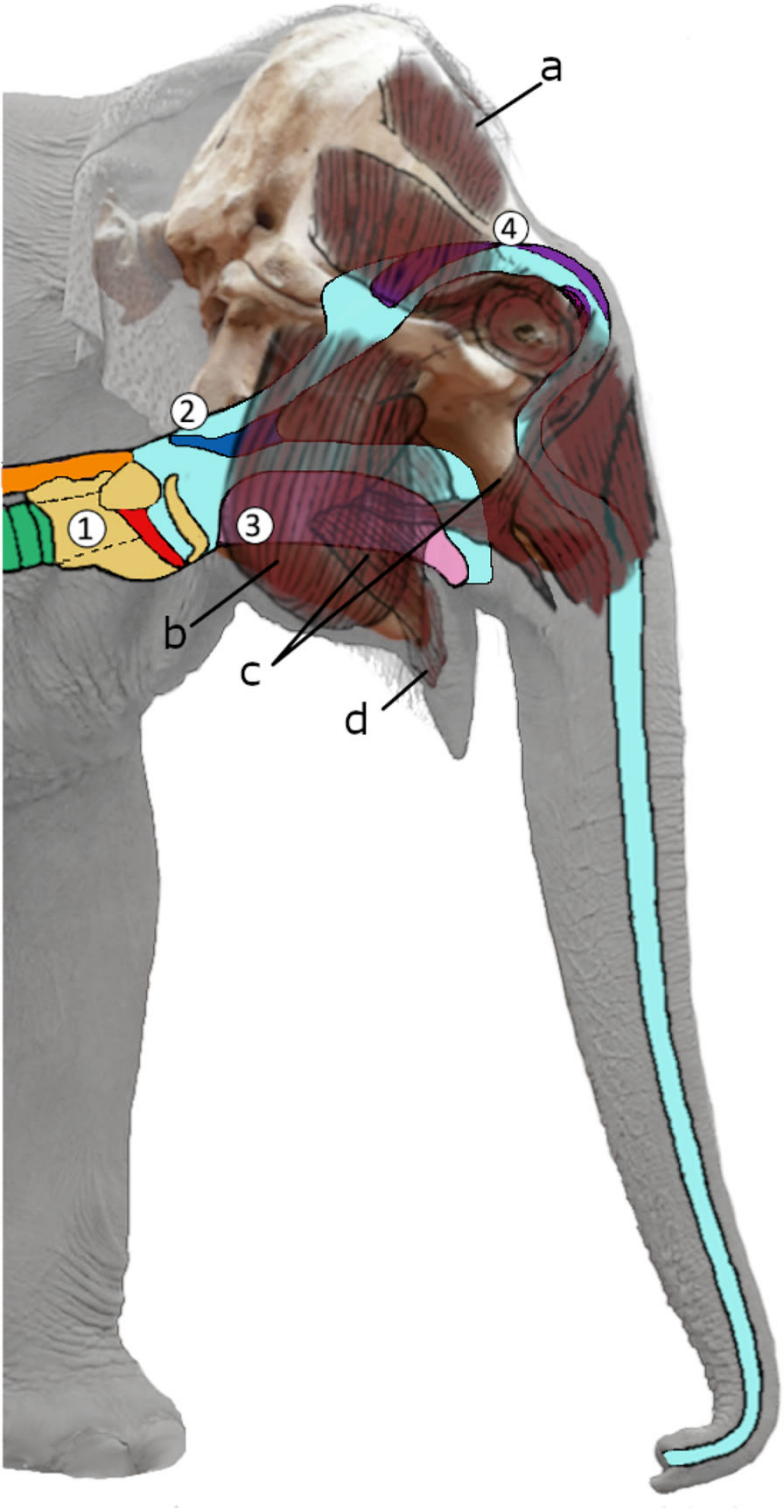
Schematic figure of vocal tract: (**1**) larynx (yellow), vocal folds (red), trachea (green), esophagus (orange), (**2**) velum (blue), (**3**) tongue (pink), (**4**) nasal cartilages (violet); facial musculature: (**a**) musculus (m.) temporalis, (**b**) m. masseter, (**c**) m. buccinator, (**d**) m. orbicularis oris. The relative position of the skull is depicted in the background

Across mammals, vocal diversity is largely bound to the bio-mechanical constraints of the vocal folds. Depending on the extent of elongation and stress tolerance, vocal folds generate fundamental frequencies (F0) spanning 2–5 octaves maximum [7]. F0 and specifically the supra-laryngeal vocal tract resonances (formants) generally decrease with increasing source and filter dimensions and hence body size (acoustic allometry, [8–10]). To enhance the vocal flexibility beyond these allometric limitations, many species developed morpho-mechanical adaptations, i.e., active muscle control of vocal folds [11], alternative vibratory tissues, or extension of their vocal tract (reviewed in [12]). Others switched to a purely aerodynamic whistle mechanism [10, 13–16]. The elephants’ high-frequency “trumpet” (F0 ~ 300–500 Hz) [17] is assumed to be produced via paired valve-shaped cartilages at the lateral sides of each of the nasal cavities set into vibration by vigorous exhalation of air [18] with no involvement of the larynx [19] (Fig. 1).

On the cognitive level, vocal plasticity in the form of the ability to learn or modify vocalizations following auditory experience (vocal production learning [20]), has a much scarcer taxonomic distribution. Bats modify innate vocalizations. A few distantly related orders of birds (songbirds, hummingbirds, and parrots) and nonhuman mammals, i.e., cetaceans, pinnipeds, and elephants, are reported to learn sounds outside their species-specific repertoires (reviewed in [21]). Interestingly, newly evolved non-laryngeal sound sources, as compared to the ancestrally shared and highly conserved larynx, are found in all those lineages yielding the canonical vocal learning species. This relation, however, has received little notice nor has it explicitly been studied [22, 23]. Indeed, at species level, the extent of vocal flexibility and sound production mechanisms are not always conclusively known.

In this context, Asian elephants are a particularly interesting species. First, they produce high-pitched squeaks with F0 reaching up to 2 kHz [24], also termed “chirps” [25, 26] or “squeals” [27]. These high-pitched sounds are absent in the naturally occurring repertoires of the African elephant species, the African savannah elephant (*Loxodonta africana*), and African forest elephant (*Loxodonta cyclotis*) [17], but similar sounds haven been reported in a case of sound imitation by a captive African elephant [28]. Second, an Asian elephant is among the two cases of mammals ever demonstrated to imitate human speech [29], the other being one harbor seal (*Phoca vitulina*) [30]. Despite this demonstration of elaborate imitative skills, the mechanisms and adaptive functions of vocal learning in the different elephant species are currently unknown and difficult to address, especially considering that their communicative systems are not yet completely understood.

In the wild, all elephant species live in highly social and vocally active matriarchal fission-fusion societies that, while differing in association patterns and group sizes, are all based on core female kin-units; males disperse when adolescent [31–33]. Although captive elephants are more often unrelated, they still form strong and enduring bonds that are reflected in high frequencies of close proximity, affiliative behavior, separation distress, and greeting upon reunion [34–36]. All three elephant species share about 8–10 call types that are suggested to be produced by the larynx and/or trunk. Their repertoires exhibit considerable flexibility in within-call-type variation and call combinations [17, 37]. All elephant species produce “rumbles” at low, partially infrasonic frequencies (< 20 Hz). In African savannah elephants, there is strong evidence that the rumble is produced laryngeally [29]*.* Rumbles can be orally as well as nasally emitted [38], expanding the available acoustic parameter space to encode information (e.g., identity, reproductive state, dominance, arousal (reviewed in [19]), sex [39], age [40], and alarm [41]).

In comparison, little is known about the higher-frequency calls. Asian elephants produce squeaks in alarming or socially arousing contexts [24–27]. Sikes (1971) [42] proposed the source of squeak production to be a valve-shaped intercommunicating canal uniting the right and left nasal passages of the trunk and associated fibrous arches 13 cm from the tip of the trunk. These structures, however, were to our knowledge only described once by Anthony and Coupin (1925) in one dissected subadult female Asian elephant [43] but not found in later dissections that aimed to look for these structures in two more specimens [44]. McKay (1973) suggested that squeaks are produced in the same way as trumpets [24], without the sound production of trumpets being conclusively known until to date. In summary, whilst the acoustic structure as well as the calling context of the squeak were broadly described, the encoded information (i.e., identity, physical, and motivational attributes of the caller) as well as the production mechanism remained unexplored.

In this study, we aim to reveal how Asian elephants produce squeaks. Comparison across taxa suggests that two mechanisms can be applied to achieve exceptionally high frequencies (even reaching into the ultrasonic range > 20 kHz). First, tissues may vibrate either in extension of the vocal folds, e.g., thin membranes as in microbats or nonhuman primates (reviewed in [45]), or distinct from vocal folds, e.g., phonic lips in the nasal passage of odontocetes [46]. Second, an aerodynamic whistle may be produced when a sound pressure wave is generated through vortex shedding of an airstream forced through a narrow orifice or over an edge [2], e.g., in the tightly constricted larynges in some rodents [13, 14], in a narrowing in the nasal vocal tract as suggested, e.g., in dholes [15] and wapiti [10], or in the pursed-lips of, e.g., walruses [47] and humans [16]. Many methods that were applied to reveal these mechanisms (e.g., high-speed stroboscopy, X-rays, heliox chamber, post-mortem examinations) are hardly feasible with elephants given their large body size, endangered status, and longevity.

We therefore applied sound visualization technology to identify in vivo whether squeaks are emitted through the trunk as previously hypothesized [24, 42] or through the mouth, as our previous observation of conspicuous facial movements led us to suspect. We conducted a detailed acoustic analysis to evaluate whether the squeak’s spectral structure is more consistent with tissue vibration or aerodynamic whistling. Further, we relate the proposed mechanisms to this call type’s potential for encoding information about attributes of the caller, i.e., identity. In combination with observations of body movement and morphology, we establish a model of squeak sound production. Our study adds insights into the functions and mechanism of the Asian and African elephants’ extensive vocal flexibility and sets the foundation for further investigations.

## Results

In each subsection, we present our own results together with anatomical or acoustic findings from previous studies. Using this approach, we progressively provide hypotheses and conclusions to build a comprehensible model of sound production.

### Facial movements and respiration during squeak production

**Fig. 2:**
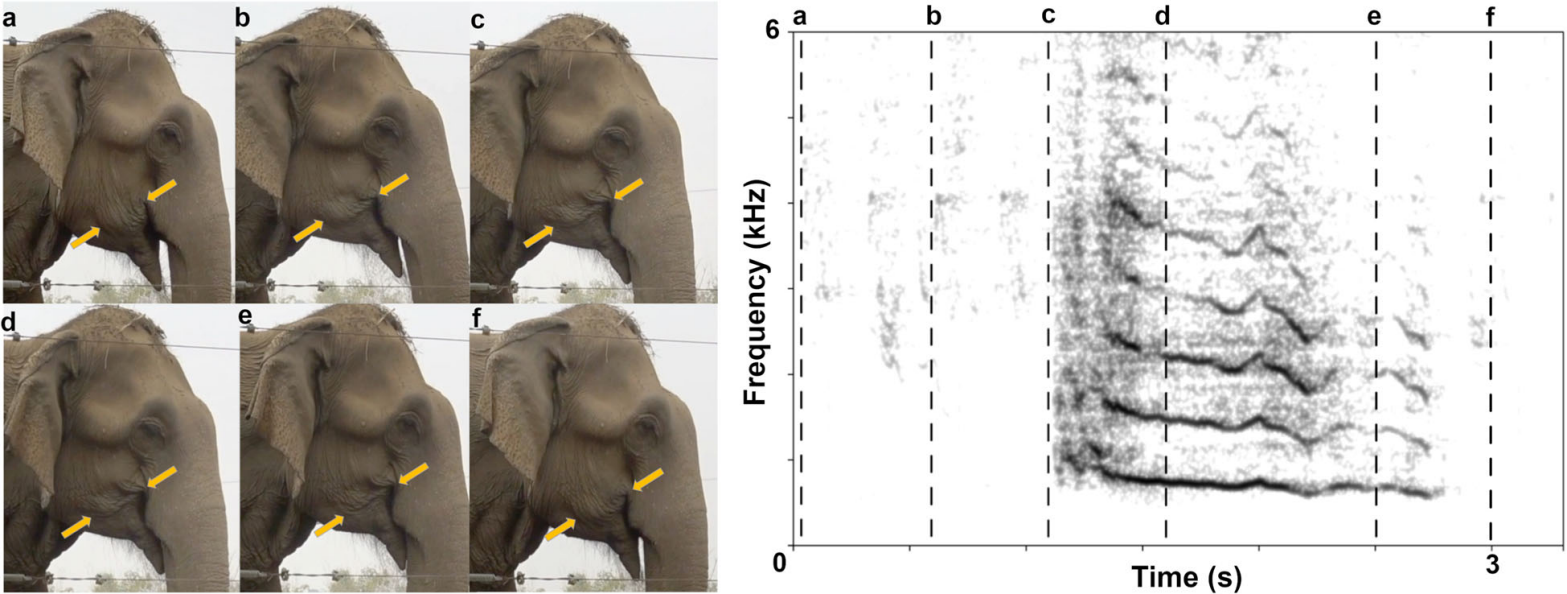
Facial movements (f, 55 years) during squeak production and the corresponding narrowband spectrogram: **a** Mouth relaxed and slightly open in resting position, **b** mouth closing in preparation of squeak production, **c** mouth fully closed and labial angles retracted at squeak onset, **d,e** cheeks depressed successively during squeak production, and **f** mouth relaxed again. Pictures were extracted from the video Additional file 1, where more subjects can be viewed squeaking


Squeak onset coincided with a conspicuous movement of oromandibular and orofacial muscles (Fig. 2 and video Additional file 1) that initially suggested their involvement in sound production. The mouth was closed, the labial angles retracted, and the cheeks successively depressed simultaneously in all subjects that squeaked (*N*_subjects_ = 21) (exception: one individual that squeaked on command and potentially used a different mechanism). Only for one elephant (Maxi, male, 50 years, details on study subjects see Additional file 2: Table S1) was it obvious that he squeaked during egression. Previous anatomical and physiological studies in elephants found that, in lacking a pleural cavity, elephants primarily breathe by contracting the diaphragm and displacing the abdominal contents. This, as opposed to thoracic expansion, makes it difficult to observe their respiration (reviewed in [48]). The closing of the mouth could suggest nasal emission. However, our observation of one elephant squeaking while its trunk was filled with water hinted at oral emission. During nasal water intake, the nasal passage is thought to be completely closed at the entrance to the skull by a combination of cartilage and muscles [18, 49] (Fig. 1), while elephants can voluntarily control their respiration, i.e., hold their breath or breathe through the mouth [48].

**Additional file 1.** Video of three elephants squeaking: 1.) begging (f, 55 years), 2.) arousal (f, 48 years), 3.) water suction and receiving handler commands (f, 11 years).

### Visualizing sound emission with an acoustic camera

**Fig. 3:**
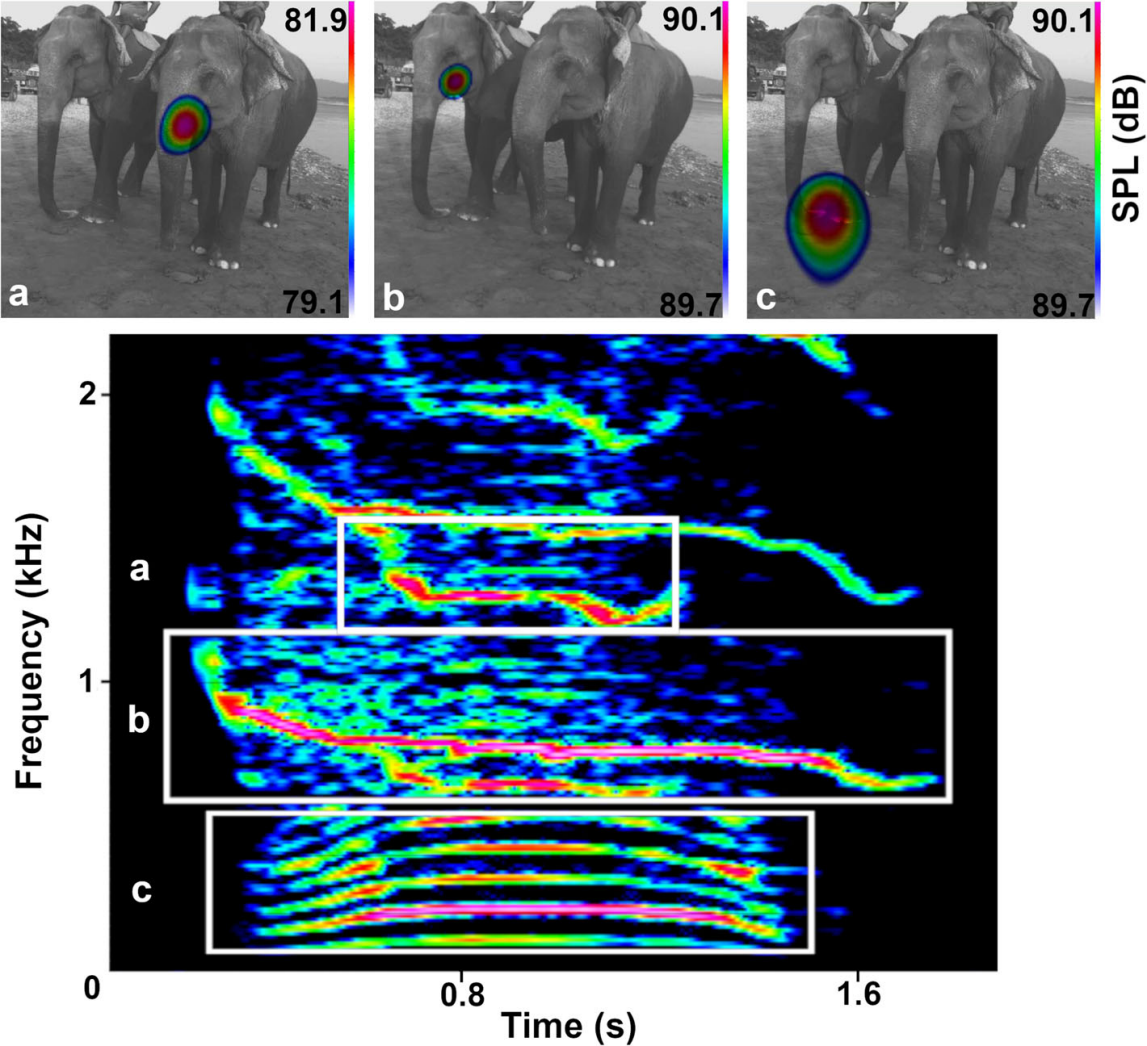
Spectrogram and acoustic camera images: **a** Squeak (2nd harmonic) orally emitted (f, 60 years). **b** Squeak (F0) orally emitted by other individual (f, 55 years). **c** Snort simultaneously uttered through the trunk


We used an acoustic camera (gfai tech) that visualizes sound by relatively color coding the effective sound pressures on the image plain based on a delay-and-sum beamforming algorithm [31]. This enabled us to clearly locate the dominant source of sound emission of squeaks at the mouth in all recorded calls (*N*_calls_ = 90, *N*_subjects_ = 3, see video Additional file 3). We also captured the simultaneous emission of a long tonal nasal snort with an oral squeak in one individual on the acoustic camera (Fig. 3 and video Additional file 4). In that female, the squeak part of this and five additional audio-recorded two-sourced biphonation calls did not differ in acoustic structure (mean F0 ± SD of squeak part = 833.20 ± 39.15 Hz, mean F0 ± SD of snort part = 103.00 ± 5.49 Hz, *N*_calls_ = 6) from her squeaks emitted solitarily (mean F0 ± SD single squeak = 908.57 ± 175.11 Hz, *N*_calls_ = 29). This contradicts any crucial involvement of the trunk in squeak sound production and implies a sound source along the oral vocal tract.

**Additional file 3.** Acoustic camera video of squeak production during begging (f,55 years).


**Additional file 4.** Acoustic camera video of a biphonation call and a squeak (left: f, 55 years, right: f, 66 years).

### Observations of mouth anatomy in living subjects and a skull

**Fig. 4:**
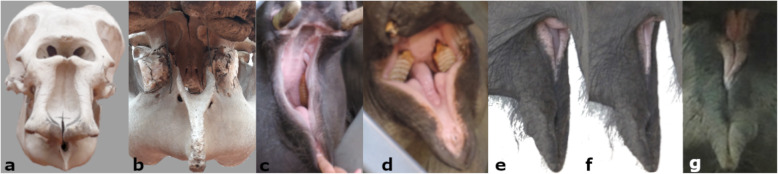
Anatomical details of mouth and lips: **a** Asian elephant skull in frontal view and **b** view into the oral cavity from below the maxilla. **c, d** Mouth opened on command in **c** m, 7 years and **d** f, 35 years. **e**, **f** Mouth closing and opening while feeding (f, 45 years), **e** tongue visible in the middle and upper lips above labial angles and **f** mouth fully closed, note the two sides of the lower lip slightly overlapping while the tip of the lip hangs loosely, images from Video S2. **g** Frontal view of closed mouth, trunk lifted during social interaction (f, 42 years)


Since the trunk obstructed the frontal and lateral view on the mouth during phonation, we observed mouth anatomy in elephants trained to open it on command, during feeding, and other activities (Fig. 4c–g). Combined with our study of an elephant skull and literature on the anatomy of elephants, this reveals the oral cavity of the Asian elephant to be relatively small, confined by the gutter-like bone structure of the lower jaw (Fig. 4a, b) and mostly filled by the tongue (Fig. 1) [18, 48–52]. While the upper lips fuse into the trunk, the lower lip is small but thick and fleshy along the labial angles towards the pointy tip and has a mucous inner surface. We observed that the mouth can be tightly closed in a longitudinal direction in which the left and right axis of the lip partly overlap (Fig. 4e–g and video Additional file 5). We suggest that the movements during phonation indicate the musculus buccinator retracting the labial angles and depressing the cheeks; its fibers passing into the musculus orbicularis oris in the lower lip (musculature described in [50]) can account for the simultaneous tension and closure of the mouth in a longitudinal direction (the very tip hangs loosely) during squeak production (Fig. 1). Combined, these observations imply that the depressing cheeks, potentially together with exhalation, generate air pressure that either produces a whistle sound by air flowing through a narrow slit of the lips or else the vibration of the tensed lips (note that the closed lips resemble the shape of vocal folds). Previous anatomical studies add that the cavity enclosed by the cheeks in a contracted state is small, but the cheeks are capable of distention [49] and thus appear suitable to create air pressure.

**Additional file 5.** Video of three elephants’ mouths during feeding (f, 48 years; f, 53 years; f, 11 years).

### Descriptive acoustic analysis

**Fig. 5:**
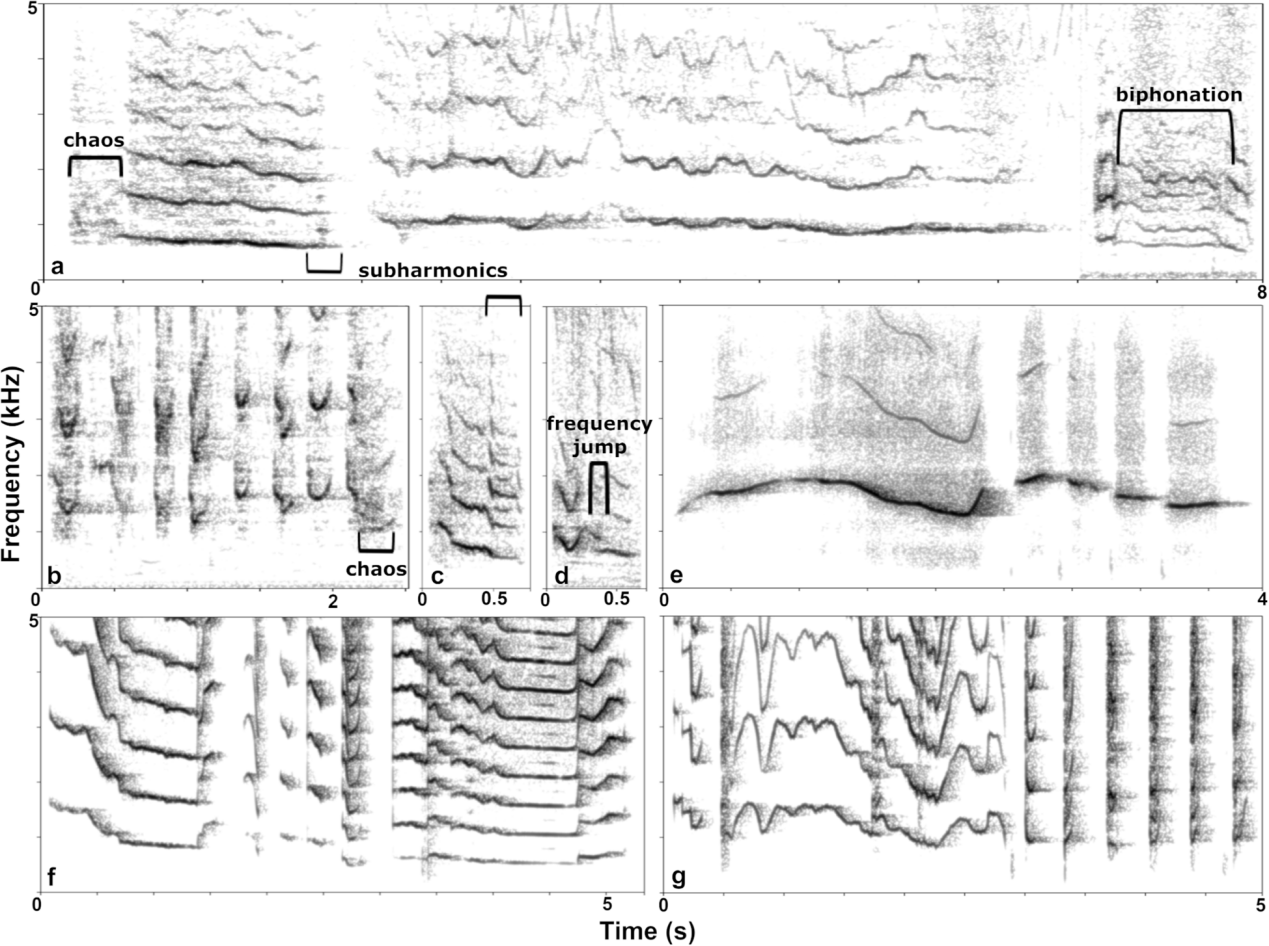
Spectrograms: **a**–**d** Squeaks from four different adult female Asian elephants demonstrating intra-individual (**a** + **b**) and interindividual (**a**–**d**) variability in spectral and temporal features and containing examples of nonlinear phenomena: **a** long squeaks that were emitted as single calls and concatenated for display (f, 55 years), **b** short squeaks that were emitted in a bout (f, 23 years); **c** f, 60 years, **d** f, 48 years, **e** the first author whistling; **f** a balloon when letting the air stream through its tensed neck; and **g** the first author buzzing her lips

With a detailed acoustic analysis, we narrowed down the potential sources: the spectral structure contradicted whistle sound production and suggested tissue vibration, yet without involvement of the vocal folds. Whistling typically produces high-frequency tonal, nearly sinusoidal, sounds in which most of the energy is concentrated in the fundamental and little or none in higher harmonics, resulting in a steep negative spectral slope (Fig. 5e) [53, 54]. We found that squeaks were indeed high-frequency vocalizations (mean F0 813.07 ± 318.72 Hz) but with a flat and at times positive spectral slope. The dominant frequency (DFR) coincided with the fundamental frequency in most but not all the calls (82.00 ± 3%, Table 1)*.* The mean differences among the amplitudes of the first harmonics were relatively small or even negative (e.g., 2nd-1st spectral peak: − 5.52 ± 6.55 dB, Table 1). We could not reliably detect patterns of energy distribution across calls above the 4th harmonic and were thus unable to find energy concentrations indicative of formant frequencies in squeaks. Formants would indicate vocal tract resonances downstream from the sound source and are clearly identifiable in elephant rumbles [31, 39]. In narrowband calls, the source frequencies may be locked on one formant [55] or simply not coincide with any resonant frequencies of the vocal tract. Here, their absence can also be interpreted to indicate that squeaks are not filtered by the vocal tract, further supporting our model of labial sound production.

**Table 1:** Descriptive statistics of parameters related to the spectral structure and temporal patterns of squeaks. The mean is calculated over the individual mean values except for those variables with a preceding “total” (calculated over total sample size)

	*N*_subject_	*N*_calls_	*N*_calls/subject_	Minimum	Maximum	Range individual means	Mean (±SD)
**General spectral structure**							
Mean F0 frequency (Hz)	10	224	10–29	291.84	2001.72	471.84–1536.76	813.07 ± 318.72
Dominant frequency (Hz)	10	238	9–34	421.90	3079.20	565.74–1609.93	978.27 ± 378.31
Percent calls per subject with DFR on 2. harmonic (%)	10	230	9–29	0.00	91.00	–	18.00 ± 3.00
HNR (dB)	7	107	7–24	1.01	26.86	3.93–14.91	11.00 ± 3.55
Percent of calls per subject with at least one NLP (%)	10	213	2–41	73.68	100	–	97.12 ± 8.27
2.-1. Spectral peak (dB)	10	209	4–24	−31.00	16.50	−15.42 - 8.47	−5.52 ± 6.55
3.-2. Spectral peak (dB)	7	78	4–16	−4.20	26.60	−14.77 - 7.52	−3.99 ± 6.69
4.-3. Spectral peak (dB)	7	31	1–10	−1.00	21.10	–	total 8.39 ± 5.92
Sound pressure level (dB)	2	10	3–7	58	96	–	total 76.22 ± 11.54
**Temporal patterns**							
Percent of calls uttered in bouts per subject (%)	15	976	12–244	52.94	100	–	79.3 ± 18.01
Number of calls per bout	15	1036 Bouts 154	12–244 Bouts 2–29	2	25	2.67–8.79	4.89 ± 2.11
Call duration (s)	13	765	13–244	0.04	4.49	0.14–2.04	0.47 ± 0.51
Interval duration (s)	13	Bouts 113 Interval 498	3–23 4–144	0.03	3.42	0.14–1.12	0.50 ± 0.32

### Nonlinear phenomena

Squeaks varied in their degree of periodicity (harmonic-to-noise ratio 1.01–26.86 dB, mean 11.00 ± 3.55 dB), and some exhibited a broadband energy distribution (maximum energy detected at 13135.1 Hz from 1 m recording distance). While most squeaks contained some tonal parts, the majority (97% ± 8, Table 1) showed at least one nonlinear phenomenon (NLP; Fig. 5a–d). In total, chaos (59%) occurred most frequently, followed by biphonation (19%) (here the two-sourced calls described above were excluded), subharmonics (12%), and sidebands (2%). Two thirds (59%) of calls exhibited two, 41% three, and 35% four and five occurrences of NLP. Frequencies of NLP-type occurrences varied considerably among individuals (Additional file 2: Table S2). The squeaks’ harmonic structure and varying degree of periodicity indicated their generation through tissue vibration, as typically found in laryngeal sounds [4].

### Correlation of fundamental frequency and caller age

**Fig. 6:**
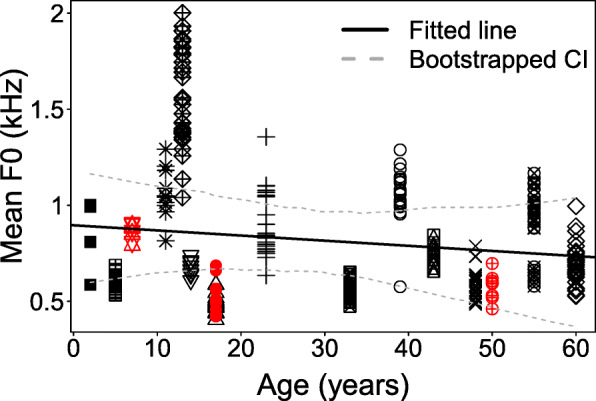
Squeak mean F0 across call (Hz) plotted against age (years). Fitted regression line (*y* = 895.8–2.670x) for 13 females (black symbols) (*N*_calls_ = 225, *N*_call/subject_ = 4–30, *χ*^2^ = 0.419, df = 4, *P* value = 0.51, Table S4), and 3 males that are not included in the model (red symbols)

For squeaks to be a laryngeal call, we would expect the F0 to decrease with age and body size, as in African elephant “rumbles” [39, 40, 56, 57]. Similarly, the F0 of whistles negatively correlated with body size and the related dimensions of the resonator in humans [16] and dogs [58]. Asian elephants continuously grow until an age of 15 years in females and 35 years in males, and gain weight until 21 and 50 years in females and males, respectively [59]. We therefore tested the relation of age as an approximate indicator of body size with squeak F0. We found no effect on F0 when testing 13 females (age 2–55 years), entering age as fixed and individuals as a random effect in a linear mixed model (*N*_calls_ = 225, *N*_call/subject_ = 4–30, *N*_subjects_ = 13, *χ*^2^ = 0.419, df = 4, *P* value = 0.51, for detailed model results see Additional file 2: Table S3). *R*^2^ for the effect of age explained only 2% of variance, but 82% percent combined with individual variance. Since we had acoustic data for only three males (two adults, one calf), we did not include them into the model, but found their squeaks to lie within the females’ F0 range. A 50-year-old male weighing 4910 kg was the largest subject of our sample, yet his F0 was comparable to that of the 7-year-old female juvenile (1865 kg) (Fig. 6). Consequently, our data contradict squeaks being generated by a whistle mechanism bound to a resonator, vocal fold vibration, or any tissue that is strongly influenced by the animal’s age, body size, and possibly sex.

### Individual discriminability and call subtype classification

We show considerable inter- and intra-individual variation in the spectral and temporal patterns of squeaks (Table 1 and Additional file 2: Table S4, Fig. 5a–d). A cross-validated discriminant function analysis (DFA) based on parameters related to the F0, its modulation, contour shape, and call duration (for a detailed description of the parameters, see Addition file 2: Table S5) [60, 61] confirmed individual discriminability. Squeaks could be classified to the correct caller with a success rate of 75%; a permutation DFA to control for nested data affirmed this result to be above chance (*N*_ind_ = 10 females, *N*_calls_ = 100, *P* value < 0.005). Apart from that, squeaks could not be categorized into meaningful call subtypes as suggested in an earlier study that did not control for individual variation [27]. *K*-means clustering explained more than 70% variance only after the number of clusters became equal to the number of subjects (*N*_subjects_ = 10).

### Comparison to human lip buzzing and a simple balloon model

In conclusion, self-sustained vibration of only a part of the lip mass remains the most likely squeak generator. This interpretation is supported by a test in which the first author artificially produced squeaks by buzzing her lips or letting the air of a balloon stream out while tensing its neck with the fingers (50 cm diameter inflated, neck 4 cm): these simple models closely matched both the spectral structure and variety of temporal patterns observed in elephant squeaks (Fig. 5f, g).

### Prevalence of squeaking individuals and behavioral context

We recorded squeaks from only 19 of 56 elephants across all age classes and sexes from both systematic and experimental data collection (see Additional file 2: Table S1). In our first study group, we combined systematic observations with separation-reunion experiments over a total of 58 days, producing 548 h of acoustic recordings among two observers: only four out of 14 elephants produced squeaks, but did so regularly. Although we did not record at night-time, our accommodations as well as those of the handler were next to the elephant enclosures, and the elephants could be heard vocalizing at night, especially the ones squeaking. In interviews, we conducted with all elephant handlers, some of whom spend decades (up to 50 years) with the elephants, they stated to never have heard a squeak from any of the elephants we were unable to record squeaking during our observation time. We recruited the additional study groups by asking handlers and facility managers to report the number and identity of squeaking elephants a priori. By playing back calls from unfamiliar elephants, we were able to reliably induce arousal and elicit squeaking in all these groups. In one additional group, squeaks were only recorded opportunistically from three out of 22 subjects, but here we did not try to elicit call emission experimentally.

Our study groups consisted of unrelated subjects or were mixed with mother-offspring units. Interestingly, in those cases where the offspring lived together with a squeaking mother, the offspring was always also found to squeak (6 dyads, see Additional file 2: Table S1). In line with previous studies, squeaks were recorded in arousing contexts, either in response to an alarming external stimulus, including smells or noises (e.g., dog, car, unfamiliar elephants in vivo, or a playback of their sounds), or during social arousal such as reassurance [62], greeting upon reunion, or in submissive reactions towards dominant conspecifics or a commanding handler [24–27] (see “Methods” for behavioral details). In all these cases, another elephant was always close by (92% within one body length, 8% within 2–5 body lengths, *N*_calls_ = 1033, *N*_subjects_ = 15). Moreover, one elephant (“Maxi,” Zürich Zoo) squeaked when greeting a handler with whom he reportedly had a very affectionate bond, but not while we observed him socializing with his conspecifics. Three elephants squeaked while begging for food. Five elephants were trained to squeak on vocal command even when separated from conspecifics, one of them (“Kreeblamduan,” Additional file 2: Table S1) even exclusively, i.e., she was not reported or observed to do so in any social context. That individual might apply yet another mechanism potentially involving the trunk because no conspicuous cheek depression was evident during phonation, but this cannot be further specified without detailed investigations.

## Discussion

### Sound production mechanism

We suggest that Asian elephants “buzz” their lips to squeak, a mechanism that—besides human brass players—has not been described elsewhere in the animal kingdom. This adds to the extraordinary vocal flexibility, especially in the modifications of the upper vocal tract (orofacial and oromandibular musculature, jaw, mouth, lips, trunk) found across elephant species [17, 19, 31, 37, 38, 63]. The proposed mechanism can account for the observed intra- and interindividual acoustic variation in squeaks, which probably involves an idiosyncratic morphology along with muscle tensioning and pressure application techniques that set only parts of the lip mass in motion. Comparably, the frequency range of trombone players depends on the airflow and the volume changes of the lips upon aperture and contraction, which mainly maintain the oscillation [3]. Only advanced players can smoothly change the pitch (lip glissando) without jumping registers [3]. Similar to vocal fold vibration [64, 65], we suggest that the nonlinear phenomena in squeaks result from changes in applied air pressure and muscle tension in the closed lips. This induces irregular vibratory patterns manifested as deterministic chaos. Different vibratory regimes occurring in parallel result in independently modulated frequencies (biphonation, here within the same source, other than the presented example of parallel nasal and oral sound production). Interactions between different vibratory regimes result in sidebands, the transition between them in frequency jumps [64, 65].

The involvement of the larynx in squeak production cannot be conclusively excluded without detailed anatomical dimensional and physio-mechanical studies of the Asian elephant’s vocal folds. Nonetheless, comparable studies render this mechanism unlikely. The 3-cm-long vocal folds of wapiti were unable to bear the tensions necessary to produce the species-specific bugles with F0 above 1.3 kHz [10, 66, 67]. Assuming comparable tissue density properties, the presumably even longer Asian elephant vocal folds (African elephants 7–10 cm) [42, 68, 69] appear even more unlikely to bear much higher tensions to reach those frequencies. We would expect the roars of Asian elephants (F0 about 500 Hz) emitted at high arousal levels and amplitudes to show the maximum achievable frequency for their laryngeal calls, yet their spectral structure significantly differs from squeaks [25–27]. Further detailed anatomical studies could go beyond evaluating our proposed model of squeak production. An across-species comparison might also broadly relate the physiological constraints of the elephant’s specific sound production organs and their call types. This would address the interesting question whether the African elephant species would be physically capable of producing squeaks, which are absent in their vocal repertoires. Differences in socio-ecological evolutionary pressures may have led to divergent anatomy of the vocal tract and communicative systems since the lineages of *Loxodonta* and *Elephas* split about 7.5 million years ago [70].

### Individual differences and proposed biological functions

The significant structural variability among individuals strongly suggests that Asian elephants can individually recognize conspecifics based on their squeaks. Further studies with larger sample sizes are needed to investigate whether attributes on caller age or sex are encoded in squeaks and whether the interindividual structural variation may be related to contextual information about calling context or arousal levels. From a functional perspective, our findings on acoustic structure and on alarming and arousing call contexts are congruent with prevalent theories of high-frequency, modulated, chaotic, and repetitive calls to signal fearful, appeasing, or conflicting motivation [71], affect, or general arousal [72] over relatively close distances [73]. NLP and the potential for biphonation from two sources, such as the squeak-snort we described, may enhance the squeaks’ unpredictability and help hinder the receiver’s habituation [65, 74] as well as boost individual acoustic distinctiveness [10, 65, 75–77]. Individual recognition may facilitate ranging, i.e., allowing the receiver to infer the sender’s distance based on the degradation of acoustic parameters [78] in dense habitats with low visibility. It may help elephants to assess the sender’s reliability in judging the alarming potential of a situation [79]. This may be important because, similar to African elephants, the discriminatory abilities of Asian elephants may increase with age [80]. Given the alarming or socially challenging contexts in which squeaks are emitted, they might serve to specifically summon up kin or unrelated but affiliated bond partners for support [81, 82]. Playback studies are required to verify whether Asian elephants can recognize individuals based on acoustic cues in squeaks [82] and to further investigate their functions.

The most striking individual difference was the absence of squeak production in the majority of our study population. Since we observed our study subjects only for restricted time periods and relied to some extent on anecdotal reports, subjects that squeak rarely or in very specific contexts might have gone unnoticed. Still, alarming, and arousing situations were observed or introduced in all groups, and those individuals who squeaked did so reliably. We conclude that not all individuals in our study were equally likely to produce squeaks in the same contexts. Elucidating the proposed functions calls for including wild populations in future studies, where we expect squeak production to be much more ubiquitous than in our captive study groups.

### Potential cognitive mechanisms

Although circumstantial, our observation that squeaks were absent in about two thirds of subjects but present in all mother-offspring pairs indicates that a non-ubiquitous genetic predisposition and/or the prerequisite of social influence from the strong mother-calf bond (or bond partners of comparable quality) may underlie squeak production. Similarly, the production of sounds to catch the attention of humans (attention-getting sounds, AG) in captive chimpanzees was reported to be socially transmitted from mothers to their offspring [83]. Overall, the likelihood of any chimpanzee to produce AG sounds did not differ among mother- and nursery-reared offspring. Chimpanzees reared by their biological mothers, however, were more likely to be concordant with their mother’s AG sound production (or lack thereof) than were the nursery-reared individuals, where social transmission among peers was proposed. In the Siberian jay (*Perisoreus infaustus*), the ability to recognize the threat a predator poses and to emit mobbing calls in this context is socially facilitated, predominantly through kin [84].

We hypothesize that the disruptions of kin and social bonds in captive Asian elephant affects the social reinforcement of the behavioral contexts in which squeaks are used. Most adult Asian elephants in captivity were wild-caught; they and their descendants are frequently translocated when sold or retired from private owners or when used in breeding programs in zoos [85]. Subsequently formed non-kin social bond may not equal the quality of family bonds in all aspects [35, 36]. Humans, however, may reinforce squeak production by specifically rewarding it or taking the place of a bond partner. One of our study subjects greeted his favored handler with squeaks, others squeaked in the context of begging. Some squeaked on command, that is in response to a conditioning stimulus from the trainer, pointing to some degree of volitional control [86]. This further underlines that Asian elephants can apparently learn to produce squeaks in different contexts [87], but the extent of their usage learning abilities remains to be tested.

The squeak production mechanism itself may also be influenced by social learning. The male Asian elephant Koshik demonstrated that in his species the upper vocal tract can be involved in learned sound production [31]. He modified laryngeal calls by putting his trunk tip into his oral cavity to imitate the formant constellation of his trainer’s commands. Three more Asian elephants within a larger captive group reportedly learned to “whistle” from each other, again by putting the trunk against the mouth [88, 89]. The acoustic descriptions of these whistles, however, did not differ from squeaks and the sound production mechanisms were not decisively investigated. Nonetheless, these reports add to our observation of the one elephant in which the conspicuous cheek depression was absent, and we suspect a trunk-based sound production. This cumulative anecdotal evidence indicates Asian elephants might use sound production mechanisms alternative to the proposed “lip buzzing” and that they might be learned. Further systematic studies are certainly needed on the social, environmental, and genetic factors influencing squeak production in Asian elephants, as well as on the underlying cognitive mechanism.

Our findings prompt considering the neuromuscular control of non-laryngeal sound production. First, arousal calls are thought to be inherently under reflex-like motor control, including the vagal stress-axis where “tensed” situations lead to tensed vocal folds and increased pulmonary pressure, typically yielding high-pitched, frequency-modulated and often chaotic calls (reviewed in [90, 91]). Finding equivalent acoustic parameters in a non-laryngeal arousal call raises the question of whether these mechanisms can be generalized. This is especially pertinent because Asian elephant squeaks might, as we argued, not be generated by reflex-like vocal motor patterns but to some extent be learned.

Second, non-laryngeal sound production mechanisms are prevalent in a range of non-vocal learning species across clades (e.g., rodents, canids, ungulates, nonhuman primates [10–15]). Interestingly, by extending the evidence for non-laryngeal sound production to include elephants, it becomes strikingly apparent that synapomorphic sound sources also occur in all the animal lineages yielding the canonical species capable of vocal production learning. Bats, which are capable of modifying innate laryngeal vocalizations (reviewed in [92]), produce their echolocation clicks by membranes controlled by laryngeal musculature [93] in all but one family: fruit bats of the genus *Rousettus* (Pteropodidae) click with their tongue (reviewed in [94, 95]). Within the complex vocal learners (i.e., those capable of imitating novel sounds, sensu [21]), the newly evolved birds’ syrinx [96] and the odontocetes’ dorsal bursae complex [46] replaced the larynx as primary sound source. Pinnipeds, in contrast, possess an entire spectrum of morpho-mechanical adaptations for sound production in addition to the larynx (reviewed in [47]). At least all complex vocal learners also share volitional respiratory control [23, 48, 97, 98], which has been proposed to be a primary gateway for enhanced vocal control [23]. This cross-species communalities offer possibilities for comparative studies of control mechanisms of innate versus learned, laryngeal, and non-laryngeal sound production, which are to date only investigated in more detail in some birds (mainly zebra finches and corvids), some nonhuman primates, and humans [86].

In the distantly related birds and humans, vocal and respiratory neuronal networks are ancestrally entwined in the brainstem, and both innate and learned vocalizations depend upon their complex coordination [86]. The volitional emission of vocalizations and vocal production learning are hierarchically controlled by forebrain regions [86]. It remains to be investigated whether and how this hierarchical neural control is similar in other vocal learning species and how it incorporates non-laryngeal sounds, and further how control circuits compare to those in species producing non-laryngeal sounds but apparently lacking the capacity for vocal production learning. Direct monosynaptic corticomotor connections are suggested to play a crucial role in vocal learning in humans (reviewed in [99]). In nonhuman primates, direct connections exist to the orofacial but not the laryngeal musculature, which parallels recent findings suggesting learned control of sound produced by the lips but a long-established absence of learned control over laryngeal sound production [99]. This illustrates how including non-laryngeal sounds in comparative bioacoustics research may help to disentangle the underlying mechanisms of different levels of vocal flexibility in the motor and cognitive domain both within and across species.

## Conclusions

We revealed that Asian elephants use a novel mechanism, “lip buzzing,” to produce vocal signals beyond the already extensive frequency range of laryngeal and trunk-based calls. This adds to our understanding of the ways in which mammals overcome the physiological limits of their sound-producing apparatus, here by including flexible use of the upper vocal tract, to widen their acoustic range available for communication. Our results further suggest that social or vocal learning processes are involved in squeak production. Following up this lead in future research would help to bridge the gap between the case studies of vocal learning in captive Asian elephants and its function and mechanisms in their natural communicative systems. Integrating non-laryngeal sound production mechanisms into broader comparative taxonomic studies will no doubt provide insights into the conundrum of the evolution of vocal learning and, ultimately, human language*.*

## Methods

### Study groups and recording periods

Calls were recorded in 2018 and 2019 across seven captive study groups, one each in Nepal, Thailand, and Switzerland, and four in Germany. Total group sizes ranged from 8 to 14 subjects. Elephants in all facilities were socially housed in subgroups (a minimum of two individuals). These consisted of families of mothers and their offspring, or of unrelated but bonded individuals, i.e., individuals that showed affiliative behaviors (such as greeting, proximity seeking behavior, separation protest) and coordinated, supportive behavior (bunching together, reassurance), or of mixed groups. Regular social interactions of friendly or tolerant subgroups were facilitated. Only some of the male elephants were kept alone at times and overnight but joined female groups on a regular basis.

In our first study group at Tiger Tops, Nepal, we recorded 12 of the 14 elephants systematically (2 elephants were kept inside the Chitwan National Park with limited access), that is two persons conducted daily observations (between 6 a.m. and 8 p.m.) alternating among groups for 54 days. Here, we conducted interviews with all their handlers with prepared questionnaires to learn how long they have been working with the elephants and if and under which circumstances they had observed their elephants squeaking, along with questions about the elephants’ origins, social bonds, and personalities. We did not find any discrepancies among the elephants reported to be squeaking by the handlers and our own observations in our first study group. Hence, for the following study groups, we asked the elephant handlers and facility managers which of their elephants were squeaking beforehand and recorded only until we had collected sufficient calls for acoustic analysis, ranging from 2 to 4 days and variable recording times (see Table S1).

Anatomical aspects were studied on one skull from the zoological collection of the University of Vienna and on the living subjects and video recordings.

### Recording context

We recorded during naturalistic observational periods (without interfering with the animals) and experimental call solicitations. In the group in Nepal, bonded individuals were briefly separated to induce vocally active greeting ceremonies upon reunion. In the study groups in Germany and Switzerland, where experimental separation and reunions were not feasible, arousal and accompanying vocalizations were triggered through noises from handlers or playbacks of unfamiliar elephants’ vocalizations. This was done with all elephants except for two males that were kept separated and where handlers had declared beforehand that these elephants did not squeak. In addition, we recorded squeaks produced on command from all elephants that were trained to do so. Those were at times singled out for such training sessions. For the group in Thailand, recordings were collected only opportunistically by HLJ (see “Acknowledgements”) from those individuals that had been indicated by the handlers and observed to squeak during her 5-month research stay, but she did not try to elicit calls in the other elephants. For all calls, we noted the behavioral context, recording circumstances and group compositions. Behaviors that are indicative of arousal even when the trigger was not obvious include “tail-raise,” “urination,” “head-raise,” “pirouette,” defensive behavior (e.g., “bunching,” that is aligning to a defensive unit, “attacking”), and finally social reassurance behavior such as frequent mutual mouth, temporal glands, and genital checks with the trunk [62, 100].

### Recording equipment

We used an omni-directional Neumann KM183 condenser microphone, modified for recording frequencies below 20 Hz (flat-recording down to 5 Hz frequency response: 5 Hz–20 kHz) with a Rycote windshield, connected to a Sound Devices 633 (at 48 kHz sampling rate and 16-bit). Only when recording the Thailand group did we use a Zoom 4Hn recorder with a built-in microphone instead. Sound pressure levels were exemplarily measured on two individuals (females aged 55 and 60 years) from 1.5–2 m distance using a NTi Audio’s Acoustilyzer AL1. For video recordings, we used a Sony Camera FD53. For playbacks to stimulate call emission, we used a JBL Charge 3 portable loudspeaker that was connected to a smartphone via bluetooth.

### Acoustic camera recordings

We recorded three squeaking elephants at Tiger Tops, Nepal, with an acoustic camera during four consecutive days in October 2018 at varying times between 6:00 am and 8:00 pm. On the acoustic camera, an array of 48 microphones is arranged on a three-armed star around a central camera for concomitant video and audio recordings [38]. The array is conically tilted forward in the direction of the sound source, creating a back-field suppression of approximately 15 dB. The array was placed about 6–8 m from the vocalizing elephant and connected to a recorder and laptop with the operating program NoiseImage. A pre-trigger and the total recordings times (max 360 s) were set beforehand.

### Acoustic camera analysis

To locate the dominant sound source, the sound pressure level (SPL) was displayed by color coding and the resulting acoustic map projected automatically onto the optical image. The effective sound pressure at point x on the image plain was calculated by a delay-and-sum beamforming algorithm. The algorithm takes into account the sum of the relative time delays or the phase shift when analyzed from the frequency domain, respectively. It considers each microphone position and compensates the run time or phase shift of the sound arriving at the microphone array (for details see [38]). NoiseImage allows adjusting the focus post-recording to locate the sound source in still images even from moving objects. Ranges of specific interest can be manually selected from the time and frequency domain to display the acoustic map in the corresponding 2D acoustic photo. We analyzed each call frame by frame (frame size 39–79 ms), either selecting the sound and its visible harmonics from the spectrogram in a modifiable rectangular selection window to exclude background noise, or specifically selecting dominant frequency contours when calls overlapped. Videos were calculated directly from the audiofile (.chl) for presentation purposes (overlap 1, framerate 25 f/s).

### Acoustic analysis

We used STx (Austrian Academy of Sciences, version 4.4.6) to calculate spectrograms (Kaiser-Bessel [8], bandwidth 22 Hz) and annotate single calls, bouts, and intervals, yielding a total of 2009 calls from 22 subjects, and to extract the durations. We analyzed detailed temporal patterns in a subset of *N* = 1036 calls from 15 individuals contributing at least three bouts, representing all age classes and sexes but excluding the context of begging and handler commands. We did not use an arbitrary predefinition of what comprises a bout based on interval duration, given the considerable interindividual variation in observed temporal patterns. Instead, we annotated call bouts where visual inspection of the spectrogram showed a clear repetitive pattern and temporal coherence, acknowledging that there is room for subjectivity.

From the spectrogram, we investigated the presence and counted the number of occurrences of nonlinear phenomena (NLP). We further calculated power spectra (Hanning, window length 40 ms, 0–15 kHz) to retrieve the amplitude at the 1st until 4th spectral peaks, which correlated with the 1st to 4th harmonic in the spectrograms and measured the frequency with the highest amplitude peak (dominant frequency). The harmonic-to-noise ratio (HNR) (time step 0.01 s, minimum pitch 150–500 Hz, silence threshold 0.1, periods per window 4.5) was measured in Praat (Boersma & Weenink, version 6.0.36) [101]. Since we did not control for the HNR sensitivity to frequency differences or varying background noises, the values should reveal only relative variation within and across individuals. Here, we excluded the calls of the one elephant from the Thailand group that was recorded with different equipment. We manually tracked the fundamental frequency contour from the spectrogram (framesize 15–80 ms, step size 1–2 ms) using a custom-designed tool in MATLAB (The MathWorks, version R2017b) [56] and extracted related acoustic parameters (described in detail in Additional file 2: Table S5) automatically.

### Statistical analysis

We conducted all statistical analyses in *R* (version 3.6.2). In descriptive analyses, we first calculated individuals’ means to control for unequal sample sizes and then the total mean over individual mean values with their standard deviations, except where the sample size was small (measurements of the sound pressure level and the difference between the 4th and 3rd spectral peak, see Table 1).

### Correlation of age and fundamental frequency

We took age as an approximate indicator of body size [59] to investigate its correlation with fundamental frequency. Our cross-sectional data on bodyweights for one group and shoulder heights in the other groups confirmed the size-age order (Additional file 2: Table S1). We applied a linear mixed model (LMM) on 13 female elephants (*N*_calls_ = 255, *N*_call/subject_ = 4–30), with the mean F0 (Hz) of squeaks as the response variable, age (years) as the fixed effect and subject as a random intercept effect using the function lmer of the package lme4 (version 1.1–21 [102]). We compared the full and the null model (intercept only) [103] using a likelihood ratio test [104]. We assessed model stability on the level of the estimated coefficients and standard deviations by excluding the levels of the random effects one at a time [105] using a function provided by Roger Mundry. This revealed stability for age but large instability regarding the intercept and random intercept (see Additional file 2: Table S3). We bootstrapped the 95% confidence intervals of the model estimates and the confidence intervals for the fitted values depicted in the plot using the function bootMer.

### Individual classification

We restricted the dataset to calls of comparably sized adult females (here above age 13) [27, 59] uttered in the context of arousal (*N*_subjects_ = 10, *N*_calls_ = 224) to control for factors other than subject identity. Due to reverberation, background noises, and NLP hindering reliable measurements, we omitted start and end fundamental frequencies and related parameters. Variable distribution was inspected through histogram and qq-plots, outliers based on *z*-scores [106]. Variables were log-transformed where it improved the distribution towards uniformity and reduced the impact of outliers. For variable reduction, 13 acoustic parameters related to F0, frequency modulation, and temporal patterns were subjected to a principal component analysis (PCA). This yielded three components explaining 84% of the variance (details see Table S6). The Kaiser-Meyer-Olkin measure above 0.5 and Bartlett’s test of sphericity (*χ*^2^ = 6046, df = 78, *p* < 0.001) justified the use of PCA. Components were determined by Kaiser’s criterion with Eigenvalues greater than 1 and scree-plot inspection. When oblique rotation was requested, only the first and second components correlated at a modest level (0.27), thus orthogonal varimax rotation was chosen [106].

To test for individual call discriminability, we ran discriminant function analyses (DFA) entering the regression scores of the three rotated components as variables on 10 randomly selected calls per individual. Since the assumption of homogeneity of variance-covariance matrices was not met, we applied quadratic discriminant analysis and report results from the leave-one-out cross-classification [107]. We conducted a permutation discriminant function analysis (pDFA) to control for the non-independency of nested data (here bouts and groups) through randomization procedures. This permutation approach is considered fairly robust against both skewed distributions and outliers [108]. We used a function written by Roger Mundry based on the function lda (linear discriminant analysis) of the R package MASS (version 7.3–51.5) but changed it to the function qda (quadratic discriminant analysis).

### Categorization

To test whether calls fall into subtypes based on their acoustic features related to the fundamental frequency (see Additional file 2: Table S5) and call duration, we applied *k*-means clustering based on Euclidean distance on the scaled variables on the same call subsets as for individual classification*.* Numbers of clusters ranged from 2 to 15, and a 50-fold randomization of initial centroids was set. Meaningful cluster aggregation was inspected by a scree-plot of within to total sum of variance and the corresponding ratio of within cluster sum of squares/total sum of squares, which equals the percentage of variance explained.

## Supplementary Information

**Additional file 2. Data Tables.** **Table S1.** Sample sizes of study subjects in each group, per sex and age category, and in total, and the percentages of individuals squeaking. **Table S2.** NLP in total and per individual calls. **Table S3.** Model results of age effects on fundamental frequency. **Table S4.** Descriptive statistics of parameters related to the fundamental frequency. **Table S5.** Description of acoustic parameters extracted from the F0 contour. **Table S6.** Rotated component (RC) loadings of variables, Eigenvalues and percent of variance explained.

## Data Availability

The datasets generated and analyzed during the current study are available from the corresponding author on reasonable request.

## Abbreviations

F0: Fundamental frequency
NLP: Nonlinear phenomenon/phenomena
HNR: Harmonic-to-noise ratio
f: Female
m: Male
