# Author Correction to: A novel theory of Asian elephant high-frequency squeak production

**DOI:** 10.1186/s12915-021-01173-3

**Published:** 2021-11-04

**Authors:** Veronika C. Beeck, Gunnar Heilmann, Michael Kerscher, Angela S. Stoeger

**Affiliations:** 1 Department of Behavioural and Cognitive Biology, Mammal Communication Lab, University of Vienna, Vienna, Austria; 2 gfai tech GmbH, Berlin, Germany

**Author Correction to: BMC Biol 19:121 (2021)**

**https://doi.org/10.1186/s12915-021-01026-z**

Following publication of the original article [1], the authors noticed that an incorrect file for Additional file 4 was captured. The corrected version of Additional file 4 is attached to this Correction and Additional file 4 has been updated in the original article accordingly.


**Additional file 4.** Acoustic camera video of a biphonation call and a squeak (left: f, 55 years, right: f, 66 years).

## References

[1] Beeck (2021). A novel theory of Asian elephant high-frequency squeak production. BMC Biol.

