# Supplementary material for: A novel theory of Asian elephant high-frequency squeak production
Source: BMC Biol. 2021 Jun 17;19:121. doi: 10.1186/s12915-021-01026-z (PMC8210382; doi:10.1186/s12915-021-01026-z)
Supplement: Supplementary file 5 — Additional file 2. Data Tables. Table S1. Sample sizes of study subjects in each group, per sex and age category, and in total, and the percentages of individuals squeaking. Table S2. NLP in total and per individual calls. Table S3. Model results of age effects on fundamental frequency. Table S4. Descriptive statistics of parameters related to the fundamental frequency. Table S5. Description of acoustic parameters extracted from the F0 contour. Table S6. Rotated component (RC) loadings of variables, Eigenvalues and percent of variance explained. [file 12915_2021_1026_MOESM2_ESM.docx]

**Additional File 2: Data tables.**

**Table S1.** Sample sizes of study subjects in each group, per sex and age category, and in total, and the percentages of individuals squeaking omitting the group where data was collected opportunistically. Subjects are categorized as non-adult when <13 years (f), < 21 years (m) and adult when >13 years (f), >21 years (m); oc: subjects that squeak on command.

| **Group**  **(age-range)** | **Total number of subjects** | | | | | **Squeaking individuals** | | **Month, year, and duration of data collection (days), and total acoustic recordings (hh:mm)** |
| --- | --- | --- | --- | --- | --- | --- | --- | --- |
|  | **Group** | **F** | **M** | **Non-adult** | **Adult** | **N** | **Name, sex, age in years (y), shoulder-height in cm or weight in kg where available, and their family ties** |  |
| Tiger Tops, Nepal  (11-60) | 14 | 14 | 0 | 2 | 12 | 4 | Kanchi (f, 11 y, 211 cm) oc; Dhibya (f, 48 y, 249 cm); Pawan (f, 55 y, 240 cm); Dipendra (f, 60 y, 249 cm) | 02-04/18 & 10/18, 58 days,  2 observers, 547:47 |
| Zoo Münster, Germany (23-53) | 5 | 4 | 1 | 0 | 5 | 2 | Corny (f, 23 y), Alexander (m, 41 y) oc | 02/19,  2 days, 01:18 |
| Zoo Leipzig, Germany (<1-46) | 9 | 6 | 3 | 2 | 7 | 2 | Mother: Trinh (f, 37 y) - Son: Voi Nam (m, 17 y) | 05/19,  2 days, 13:00 |
| Tierpark Berlin, Germany  (3-39) | 7 | 6 | 1 | 4 | 3 | 1 | Astra (f, 39 y) | 02/19 & 05/19,  2 days, 01:05 |
| Zoo Cologne, Germany  (3-50) | 13 | 7 | 6 | 6 | 7 | 3 | Mother: Marlar (f, 13 y) - Son: Moma (m , 7 y); Kreeblamduan (f, 35y) oc | 09/19,  2 days, 05:24 |
| Zürich, Switzerland (2-50) | 8 | 6 | 2 | 4 | 4 | 7 | Mother: Ceyla-Himali (f, 44 y, 3315 kg)- Daughter: Farha (f, 14 y, 2875 kg) - Granddaughter: Ruwani (f, 2 y, 1000 kg); Mother: Indi (f, 33 y, 3010 kg) - Daughter: Chandra (f, 17 y, 3035 kg) - Daughter: Omysha (f, 5 y, 1865 kg); Maxi (m, 50 y, 4910 kg): fathered all offspring except for Ruwani | 07/19,  4 days, 21:19 |
| **Total (2-53)** | **56** | **43** | **13** | **18** | **38** |  | | |
| **Number Squeaking** | **19** | **15** | **4** | **4** | **11** |  |  |  |
| **Percentage Squeaking (%)** | **33.9** | **34.8** | **30.7** | **22.2** | **28.9** |  |  |  |
| GTAEF, Thailand  (2-62) | 22 | 19 | 3 | 1 | 21 | 3 | Pumpui (f, 43 y), Yui (f, 28 y) oc, Poonlarb (f, 34 y) oc | 07/19, 4 days, 00:08 (opportunistic) |

**Table S2.** Percentages of calls with non-linear phenomena (NLP) and percentages of single NLP types NLP in total and per individual

|  | Har-monic | Calls with NLP | N Calls  Sum | Chaos | Bi-phonation | Frequency-jump | Side-bands | Sub-harmonics | N NLPs  Sum |
| --- | --- | --- | --- | --- | --- | --- | --- | --- | --- |
| Astra | 0% | 100% | 23 | 51% | 49% | 0% | 0% | 0% | 43 |
| Chandra | 0% | 100% | 2 | 0% | 0% | 100% | 0% | 0% | 2 |
| Corny | 0% | 100% | 25 | 76% | 3% | 0% | 9% | 12% | 33 |
| Dhibya | 26% | 74% | 19 | 61% | 17% | 11% | 6% | 6% | 18 |
| Dipendra | 2% | 98% | 41 | 83% | 4% | 8% | 2% | 2% | 48 |
| Farha | 0% | 100% | 4 | 0% | 50% | 0% | 25% | 25% | 4 |
| Indi | 0% | 100% | 16 | 83% | 17% | 0% | 0% | 0% | 18 |
| Marlar | 0% | 100% | 36 | 63% | 27% | 11% | 0% | 0% | 56 |
| Pawan | 0% | 100% | 32 | 29% | 9% | 16% | 0% | 46% | 56 |
| Pumpui | 0% | 100% | 15 | 58% | 27% | 0% | 4% | 12% | 26 |
| **Sum** | **3%** | **97%** | **213** | **59%** | **19%** | **8%** | **2%** | **12%** | **304** |

**Table S3.** Model results of age effects on fundamental frequency: Estimates and standard errors together with confidence limits, results of the likelihood ratio test, and the range of estimates obtained when dropping levels of the random effect one at a time, and below the estimated standard deviation for the contribution of the random effect and residual standard deviation.

|  | **Estimate** | **Std. Error** | **t value** | **CI low** | **CI up** | **χ2** | **df** | **P** | **min** | **max** |
| --- | --- | --- | --- | --- | --- | --- | --- | --- | --- | --- |
| Intercept | 895.786 | 149.997 | 5.972 | 602.083 | 1185.420 |  |  |  | 757.58 | 971.853 |
| age | -2.670 | 4.446 | -0.601 | -11.019 | 5.914 | 0.419 | 4.00 | 0.517 | -4.425 | 0.021 |
|  |  |  |  |  |  |  |  |  |  |  |
| **random effect** | |  |  |  |  |  |  |  |  |  |
| **term** | **effect** | **sd** |  |  |  |  |  |  |  |  |
| individual | intercept | 298,46 |  |  |  |  |  |  |  |  |
| residual | - | 139.46 |  |  |  |  |  |  |  |  |

**Table S4.** Parameters related to the fundamental frequency (F0) on N_subjects_ =10, N_calls_=224, and 10-29 calls per subject.

|  | **Minimum** | **Maximum** | **Range individual means** | **Mean (±SD)** |
| --- | --- | --- | --- | --- |
| **F0 related parameters** |  |  |  |  |
| Minimum frequency (Hz) | 264.04 | 1968.09 | 343.31-1441.53 | 659.02 (±336.90) |
| Maximum frequency (Hz) | 328.06 | 2464.11 | 596.23-1732.81 | 977.17 (±388.05) |
| Frequency range (Hz) | 20.00 | 1115.12 | 143.79-729.69 | 318.16 (±163.58) |
| Start frequency (Hz) | 320.05 | 2138.10 | 567.72-1656.28 | 927.63 (±324.81) |
| Mid frequency (Hz) | 264.04 | 2222.10 | 468.83-1522.27 | 824.29 (±307.70) |
| Finish frequency (Hz) | 280.03 | 2126.10 | 356.31-1576.20 | 703.48 (±382.55) |
| Mean 1st third (Hz) | 316.05 | 2153.83 | 553.96-1600.85 | 885.61 (±309.47) |
| Mean 2nd third (Hz) | 275.47 | 2177.57 | 469.75-1527.96 | 819.92 (±309.04) |
| Mean 3rd third (Hz) | 286.71 | 2031.29 | 392.26-1562.68 | 736.10 (±354.31) |
| Median frequency (Hz) | 288.05 | 1996.09 | 469.21-1549.67 | 817.52 (±317.87) |
| **F0 contour and modulation related parameters** |  |  |  |  |
| Maximum by mean frequency | 1.01 | 1.71 | 1.08-1.33 | 1.21 (±0.09) |
| Mean by minimum frequency | 1.01 | 3.55 | 1.06-2.17 | 1.33 (±0.32) |
| Maximum frequency location | 0.00 | 1.00 | 0.01-0.57 | 0.22 (±0.21) |
| Minimum frequency location | 0.00 | 1.00 | 0.37-0.99 | 0.76(±0.02) |
| Start slope | -17193.61 | 3273.07 | -3935.29-622.57 | -1684.11 (±1478.24) |
| Middle slope | -13782.81 | 2613.91 | -3998.810-758.21 | -1115.07(±1525.43) |
| Final slope | -5261.74 | 10975.38 | -3648.42-2019.88 | -801.58 (±1153.44) |
| Time from min. to max. freq. (sec) | 0.12 | 1.00 | 0.55-0.90 | 0.74 (±0.14) |
| Jitter factor | 0.47 | 14.65 | 1.38-5.34 | 2.87 (±1.29) |
| Frequency variability index | 0.00 | 1.51 | 0.03-0.82 | 0.24(±0.24) |
| Inflection factor | 0.00 | 0.54 | 0.13-0.35 | 0.23 (±0.06) |
| COFM | 0.00 | 0.50 | 0.02-0.17 | 0.06(±0.04) |

**Table S5.** Description of Acoustic parameters extracted from the fundamental frequency contour

| **Acoustic Parameter** | **Description** |
| --- | --- |
| **F0 related parameters** | |
| Minimum F0 (Hz) | Minimum of fundamental frequency |
| Maximum F0 (Hz) | Maximum of fundamental frequency |
| F0 range (Hz) | Maximal fundamental frequency minus minimal fundamental frequency |
| Start F0 (Hz) | Fundamental frequency at the onset of the call |
| Middle F0 (Hz) | Frequency at the temporal middle of the call |
| Finish F0 (Hz) | Fundamental frequency at the end of the call |
| F0 Mean 1^st^ third (Hz) | Mean frequency of the first third of the call |
| F0 Mean 2^nd^ third (Hz) | Mean frequency of the second third of the call |
| F0 Mean 3rd third (Hz) | Mean frequency of the third of the call |
| F0 Median frequency (Hz) | Median of all the frequencies of the measured harmonic |
| **F0 contour and modulation related parameters** | |
| Maximum F0 by mean F0 | Calculated as maximum frequency divided by mean frequency. |
| Mean F0 by minimum F0 | Calculated as mean frequency divided by minimum frequency |
| Maximum F0 location | Location of maximum frequency given as percentage of duration |
| Minimum F0 location | Location of minimum frequency given as percentage of duration |
| F0 Start slope | Calculated as (frequency 20-frequency 1)/(Time 20-Time 1) |
| F0 Middle slope | Calculated as (frequency 40-frequency 20)/(Time 40-Time 20) |
| F0 Final slope | Calculated as (frequency 60-frequency 40)/(Time 60-Time 40) |
| Time from min. to max. F0 (sec) | Temporal distance from the minimum frequency to the maximum frequency |
| Jitter factor [1] | Calculated variable that represents a weighted measure of the amount of frequency modulation by calculating the sum of the absolute value of the difference between two sequential frequencies divided by the mean frequency. The sum result is then divided by the total number of points measured minus 1 and the final value is obtained by multiplying it by 100. |
| Frequency variability index [1] | Calculated variable that represents the magnitude of frequency modulation across a call computed by dividing the variance in frequency by the square of the average frequency of a rumble and then multiplying the value by 10. |
| Inflection factor | Percentage of points showing a reversal in slope |
| Coefficient of Frequency Modulation (COFM) [2] | Calculated variable that represents the amount and magnitude of frequency modulation across a rumble computed by summing the absolute values of the difference between sequential frequencies divided by 10,000. |

**Table S6.** **Rotated component (RC) loadings of variables**, **Eigenvalues and percent of variance explained**: Component values were extracted through regression after varimax rotation of principal components. Bold: loadings > 0.4.

| **Acoustic parameters** | **RC1** | **RC2** | **RC3** |
| --- | --- | --- | --- |
| log max F0 | **0.99** | 0.03 | 0.07 |
| log F0 mean1st third | **0.98** | 0.07 | -0.07 |
| log mean F0 | **0.96** | 0.28 | 0.00 |
| log F0 mean 2nd third | **0.94** | 0.23 | 0.04 |
| log F0 mean3rd third | **0.84** | 0.52 | 0.03 |
| log F0 min | **0.78** | 0.59 | -0.02 |
| log Frequency Variability Index | -0.18 | **-0.93** | 0.10 |
| log Jitter Factor | -0.35 | **-0.83** | 0.00 |
| F0 Final Slope | 0.27 | **0.70** | 0.03 |
| F0 Middle Slope | 0.12 | **0.66** | **0.43** |
| log F0 duration | 0.05 | -0.07 | **0.91** |
| log COFM | 0.34 | **-0.58** | **0.67** |
| F0 Start Slope | -0.15 | 0.22 | **0.63** |
| **Eigenvalue** | **5.41** | **3.62** | **1.88** |
| **% Variance explained** | **0.42** | **0.28** | **0.14** |
